# G2019S LRRK2 Increases Stress Susceptibility Through Inhibition of DAF-16 Nuclear Translocation in a 14-3-3 Associated-Manner in *Caenorhabditis elegans*

**DOI:** 10.3389/fnins.2018.00782

**Published:** 2018-11-07

**Authors:** Simei Long, Wenyuan Guo, Sophie Hu, Fengjuan Su, Yixuan Zeng, Jinsheng Zeng, Eng-King Tan, Christopher A. Ross, Zhong Pei

**Affiliations:** ^1^Department of Neurology, National Key Clinical Department and Key Discipline of Neurology, Guangdong Provincial Key Laboratory of Diagnosis and Treatment of Major Neurological Diseases, The First Affiliated Hospital, Sun Yat-sen University, Guangzhou, China; ^2^Department of Neurology, The First Affiliated Hospital, Guangzhou Medical University, Guangzhou, China; ^3^Cumming School of Medicine, University of Calgary, Calgary, AB, Canada; ^4^Shenzhen Second People’s Hospital, The First Affiliated Hospital of Shenzhen University, Shenzhen, China; ^5^Department of Neurology, Singapore General Hospital, Singapore, Singapore; ^6^National Neuroscience Institute, Singapore, Singapore; ^7^Duke-NUS Graduate Medical School, Singapore, Singapore; ^8^Division of Neurobiology, Department of Psychiatry–Departments of Neuroscience, Neurology, and Pharmacology, Johns Hopkins University School of Medicine, Baltimore, MD, United States

**Keywords:** Parkinson’s disease, G2019S LRRK2, stress, 14-3-3, daf-16, *Caenorhabditis elegans*

## Abstract

Mutations in *leucine-rich repeat kinase 2* (*LRRK2*) are common causes of familial Parkinson’s disease (PD). Oxidative stress plays a key role in the pathogenesis of PD. Mutations in LRRK2 have been shown to increase susceptibility to oxidative stress. To explore mechanisms underlying susceptibility to oxidative stress in LRRK2 mutants, we generated stable *Caenorhabditis elegans* (*C. elegans*) strains in which human LRRK2 proteins including wild type LRRK2 (WT), G2019S LRRK2 (G2019S), and G2019S-D1994A kinase-dead LRRK2 (KD) were expressed in all neurons. Human 14-3-3 β was injected into LRRK2 transgenic worms to allow co-expression of 14-3-3 β and LRRK2 proteins. We found that G2019S transgenic worms had increased sensitivity to stress (heat and juglone treatment) and impaired stress-induced nuclear translocation of DAF-16. In addition, G2019S inhibited *ftt2* (a *14-3-3* gene homolog in *C. elegans*) knockdown-associated nuclear translocation of DAF-16. Comparably, overexpression of human 14-3-3 β could attenuate G2019S-associated toxicity in response to stress and rescued G2019S-mediated inhibition of *sod-3* and *dod-3* expression. Taken together, our study provides evidence suggesting that 14-3-3-associated inhibition of DAF-16 nuclear translocation could be a mechanism for G2019S LRRK2-induced oxidative stress and cellular toxicity. Our findings may give a hint that the potential of 14-3-3 proteins as neuroprotective targets in PD patients carrying LRRK2 mutations.

## Introduction

Parkinson’s disease (PD, [MIM: 168600]) is a neurodegenerative disorder caused by genetic and environmental factors. A substitution of serine for glycine at position 2019 (G2019S) in the kinase domain of leucine-rich repeat kinase 2 (LRRK2, [MIM: 609007]) represents the most prevalent genetic mutation in PD (Zimprich et al., 2004; Di Fonzo et al., 2005; Gilks et al., 2005; Nichols et al., 2005). Furthermore, oxidative stress is also believed to play an important role in the pathogenesis of PD (Halliwell, 2006; Gandhi and Abramov, 2012), as elevated levels of reactive oxygen species (ROS) have been implicated as a pathological feature of PD. For example, G2019S LRRK2 causes uncoupling of mitochondrial oxidative phosphorylation (Mortiboys et al., 2010; Papkovskaia et al., 2012) which consequently promotes ROS accumulation and neurodegeneration (Lu et al., 2004; Angeles et al., 2011; Lebel et al., 2012). While G2019S LRRK2 and stress are involved in the pathogenesis of PD, the underlying pathway linking these processes is unknown.

DAF-16, a homolog of mammalian FoxO (forkhead box O subclass of transcription factors), is an important transcriptional regulator of genes that rapidly respond to and neutralize the effects of oxidative stress, such as superoxide dismutase (McElwee et al., 2003). Translocation of DAF-16 from cytoplasm into the nucleus is a key step for its transcription factor activity. Moreover, 14-3-3 is a key regulator of DAF-16 nuclear translocation. 14-3-3 proteins can bind to phosphorylated FoxO in mammalian cells (Brunet et al., 1999; Durocher et al., 2000; Obsil et al., 2003) and phosphorylated DAF-16 in *C. elegans* (Cahill et al., 2001), and this binding leads to retention of forkhead proteins in the cytoplasm, thereby rendering them inactive. In parallel, 14-3-3 proteins are also required for SIR-2.1-induced transcriptional activation of DAF-16 and stress resistance (Berdichevsky et al., 2006).

The family of 14-3-3 proteins comprises evolutionarily conserved modulator proteins that regulate multiple signaling pathways through binding to specific Ser/Thr-phosphorylated motifs on target proteins (Tzivion and Avruch, 2002; Tzivion et al., 2006; Morrison, 2009). This protein family includes seven isoforms in mammals (Ichimura et al., 1988; Martin et al., 1993) and two (PAR-5 and FTT-2) in *C. elegans* (Wang and Shakes, 1996). 14-3-3s have been implicated in the pathogenesis of several neurodegenerative diseases including PD, Alzheimer’s disease, Huntington’s disease and amyotrophic lateral sclerosis. In PD, markedly low levels of 14-3-3 were detected in human PD brain. Disruption of 14-3-3 has been shown to mediate toxicity while overexpression of 14-3-3 is protective via multiple mechanisms such as inhibition of apoptosis and attenuation of protein aggregation in both genetic and toxic models of PD.

14-3-3 proteins have been shown to interact with wild type LRRK2 and several PD-associated LRRK2 mutants including R1441C, R1441G, R1441H, Y1699C, and I2020T, but not G2019S, which has been shown to disrupt the interaction with 14-3-3 proteins (Dzamko et al., 2010; Nichols et al., 2010; Li et al., 2011). Disruption of 14-3-3 protein expression and function has been recently implicated in PD pathogenesis (Slone et al., 2015). Furthermore, a strong neuroprotective effect of enhanced 14-3-3 expression has been shown in multiple cellular and animal models of PD (Yacoubian et al., 2010). Collectively, 14-3-3 proteins play an important role in LRRK2 mutant-linked Parkinsonism.

We hypothesized that G2019S LRRK2 reduces stress resistance by inhibiting DAF-16 nuclear translocation, which may be mediated by *ftt-2*. G2019S disrupts and indirectly decreases the stress response system by increasing sensitivity to stress through DAF-16. 14-3-3 β is a potential target that can rescue the loss of stress resistance ability. To investigate whether 14-3-3 protein can regulate G2019S LRRK2-induced toxicity in the current study, we generated *C. elegans* strains expressing human wild type LRRK2, G2019S LRRK2 and G2019S D1994A kinase-dead (KD) LRRK2, and injected 14-3-3 β into these LRRK2 transgenic worms. We discovered that pan-neuronal expression of G2019S LRRK2 caused defects in stress resistance and impaired stress-induced or 14-3-3 protein-associated DAF-16 nuclear translocation in *C. elegans*. We also investigated G2019S LRRK2-induced defects in *sod-3* and *dod-3* mRNA expression by modulating DAF-16 localization. Our data suggest that human 14-3-3 β [MIM: 601289] protein could rescue G2019S LRRK2-associated toxicity in response to stress.

## Results

### G2019S LRRK2 Enhanced Toxicity in Response to Environmental Stress and Impaired Stress-Induced DAF-16 Nuclear Translocation

**FIGURE 1:**
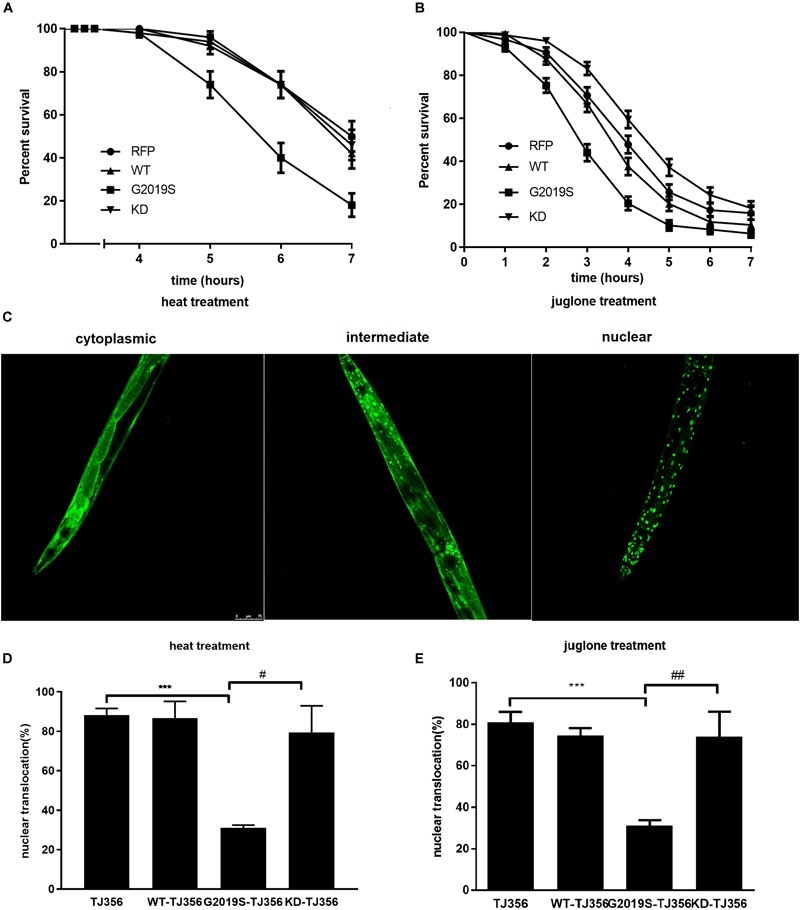
G2019S LRRK2 increased sensitivity to stress and impaired stress-induced DAF-16 nuclear translocation. **(A)** Nematode expressing G2019S LRRK2 increased sensitivity to heat stress. The RFP strain served as a control. **(B)** Nematode expressing G2019S LRRK2 increased sensitivity to oxidative stress (juglone treatment). The RFP strain served as a control. **(C)** Three different status of DAF-16 localization (cytoplasm and nucleus and both). The worm is the TJ356 strain. Scale bar = 75 μm. **(D)** Quantification of stress-induced DAF-16 nuclear translocation in adult synchronized worms. After reaching adulthood, the worms were exposed to heat stress (35°C) or juglone treatment (400 μM) for 1 h and counted for the presence of nuclear or cytoplasmic DAF16::GFP. Intermediate status and nuclear status of DAF-16 were counted as nuclear DAF-16. Error bars indicate SEM. ^∗∗∗^*P* < 0.001, represent TJ356 vs. G2019S-TJ356. ^#^*P* < 0.05, represent G2019S-TJ356 vs. KD-TJ356. **(E)** Quantification of stress-induced DAF-16 nuclear translocation in adult synchronized worms. After they reached adulthood, the worms were exposed to juglone (400 μM) for 1 h and counted for the presence of nuclear or cytoplasmic DAF16::GFP. Intermediate status and nuclear status of DAF-16 were counted as the nuclear DAF-16. Error bars indicate SEM. ^∗∗∗^*P* < 0.001, represent TJ356 vs. G2019S-TJ356. ^##^*P* < 0.01, represent G2019S-TJ356 vs. KD-TJ356.

We generated *C. elegans* strains expressing human wild type LRRK2, G2019S LRRK2 and G2019S D1994A KD LRRK2 in all neurons to investigate the responses of different LRRK2 strains to two different kinds of stress conditions: thermal and oxidative (Supplementary Figures S1, S2 and Supplementary Table S1). A strain expressing red fluorescent protein (RFP) alone served as a control. Analysis of sensitivity in response to stress was conducted in age-synchronized populations of nematodes. In the present study, all strains remained alive during the first 3 h of heat stress. However, control worms exhibited 50.0% ± 7.1% survival in response to heat stress for 7 h, while survival rates of WT, G2019S and KD transgenic worms were 42.0% ± 7.0%, 18.0% ± 5.4%, and 46.0% ± 7.0%, respectively. Compared with the control strain, WT showed a similar result, but G2019S significantly decreased the survival rate (Figure 1A, *p* < 0.01); whereas, KD could rescue G2019S-mediated loss of heat resistance (Figure 1A, *p* < 0.05). Meanwhile, similar results were achieved in oxidative stress experiments. RFP control worms exhibited 15.9% ± 3.0% survival after exposure to juglone for 7 h, while survival rates of WT, G2019S and KD transgenic worms were 10.4% ± 2.6%, 6.4% ± 2.0%, and 18.3% ± 3.2%, respectively. Compared with the control strain, WT showed similar results, but G2019S significantly decreased the survival rate (Figure 1B, *p* < 0.01); whereas, KD could rescue G2019S-mediated loss of juglone resistance (Figure 1B, *p* < 0.01).

DAF-16, a FoxO transcription factor, is a key player in stress resistance in *C. elegans*. Translocation of DAF-16 from the cytoplasm into the nucleus is necessary for its transcriptional activity (Henderson and Johnson, 2001; Lin et al., 2001). A *C. elegans* strain expressing DAF-16::GFP (TJ356) was used to investigate the role of DAF-16 in G2019S-mediated loss of stress resistance. This strain has been shown to respond to environmental stress by displaying DAF-16 nuclear translocation. Three different status of DAF-16 were show in Figure 1C. To examine nuclear translocation of DAF-16 in different LRRK2 strains, LRRK2 strains were crossed with the TJ356 strain to generate WT-TJ356, G2019S-TJ356 and KD-TJ356 strains. Numbers of worms exhibiting DAF-16 nuclear translocation was calculated 60 min after heat stress or juglone treatment. DAF-16 was predominantly localized in the cytoplasm in both WT-TJ356 and G2019S-TJ356 strains under normal conditions (data not shown). However, in response to stress, DAF-16 was translocated from the cytoplasm into the nucleus in most of the TJ356 control strain. In contrast, nuclear translocation of DAF-16 was moderate in the G2019S-TJ356 strain (Figure 1D, *p* < 0.01 for heat treatment; Figure 1E, *p* < 0.01 for juglone treatment). However, KD could rescue G2019S-mediated inhibition of DAF-16 nuclear translocation in response to heat stress (Figure 1D, *p* < 0.01) or juglone (Figure 1E, *p* < 0.01).

Under stress, percentages of worms exhibiting nuclear translocation of DAF-16 were similar among WT-TJ356, KD-TJ356 and TJ356 strains (heat treatment: TJ356, 88.2% ± 1.4%; G2019S-TJ356, 31.0% ± 1.0%; WT-TJ356, 86.7% ± 4.9%; KD-TJ356, 79.5% ± 9.5%; juglone treatment: TJ356, 81.0% ± 4.9%; G2019S-TJ356, 31.0% ± 2.7%; WT-TJ356, 74.0% ± 3.5%; KD-TJ356, 74.0% ± 12.0%). Thus, our data suggest that G2019S blocks DAF-16 nuclear translocation under stress conditions.

### G2019S LRRK2 Inhibited mRNA Expression of Stress-Resistance Genes

**FIGURE 2:**
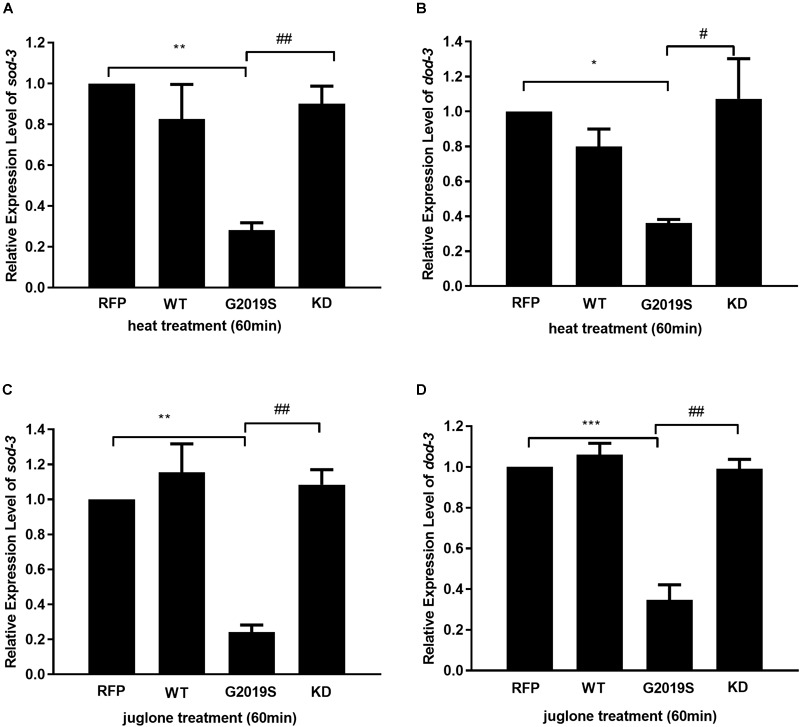
G2019S LRRK2 induced defect in mRNA expression of stress-resistance genes. **(A)** Expression of *sod-3* in transgenic strains after heat treatment for 60 min. Nematodes expressing G2019S showed reduced expression of *sod-3* (^∗∗^*p* < 0.01 versus RFP strain) whereas KD could rescue G2019S-mediated deficit in expression of *sod-3* (^##^*p* < 0.01 versus G2019S strain). **(B)** Expression of *dod-3* in transgenic strains after heat treatment for 60 min. Nematodes expressing G2019S showed reduced expression of *dod-3* (^∗^*p* < 0.05 versus RFP strain) whereas KD could rescue G2019S-mediated deficit in expression of *dod-3* (^#^*p* < 0.05 versus G2019S strain). **(C)** Expression of *sod-3* in transgenic strains after juglone treatment for 60 min. Nematodes expressing G2019S LRRK2 showed reduced expression of *sod-3* (^∗∗^*p* < 0.01 versus RFP strain) whereas KD could rescue G2019S LRRK2-associated defect in mRNA expression of *sod-3* (^##^*p* < 0.01 versus G2019S strain). **(D)** Expression of *dod-3* in transgenic strains after juglone treatment for 60 min. Nematodes expressing G2019S LRRK2 showed reduced expression of *dod-3* (^∗∗∗^*p* < 0.001 versus RFP strain) whereas KD could rescue G2019S LRRK2-associated defect in mRNA expression of *dod-3* (^##^*p* < 0.01 vs. G2019S strain).

DAF-16 controls redox metabolism by regulating the expression of anti-oxidative stress molecules and lifespan-associated genes, such as *sod-3* and *dod-3*. In response to heat stress, we found reduced expression levels of *sod-3* and *dod-3* in the G2019S transgenic *C. elegans* strain compared with controls; notably, both genes are under the regulation of DAF-16. As shown in Figures 2A,B, in response to heat stress, the G2019S transgenic strain inhibited mRNA expression of *sod-3* (0.28 ± 0.04-fold, *p* < 0.01 vs. control) and *dod-3* (0.36 ± 0.02-fold, *p* < 0.05 vs. control), while other LRRK2 transgenic strains showed no significant difference from the control strain [*sod-3* expression (WT: 0.83 ± 0.17, KD: 0.90 ± 0.09); *dod-3* expression (WT: 0.80 ± 0.10, KD: 1.07 ± 0.23)].

Similarly, we examined the mRNA expression levels of *sod-3* and *dod-3* of all strains in response to juglone. Compared with the control strain, the G2019S transgenic strain significantly reduced expression levels of *sod-3* and *dod-3*. As shown in Figures 2C,D, in response to juglone, the G2019S transgenic strain inhibited mRNA expression of *sod-3* (0.24 ± 0.04-fold, *p* < 0.01 vs. control) and *dod-3* (0.24 ± 0.04-fold, *p* < 0.001 vs. control), while other LRRK2 transgenic strains showed no significant difference from the control strain [*sod-3* expression (WT: 1.15 ± 0.16, KD: 1.08 ± 0.09); *dod-3* expression (WT: 1.06 ± 0.05, KD: 0.99 ± 0.05)]. Thus, our data suggest that G2019S modulates the regulation of stress-resistance genes.

### G2019S LRRK2 Impaired DAF-16 Nuclear Translocation in a 14-3-3 Protein-Dependent Manner

**FIGURE 3:**
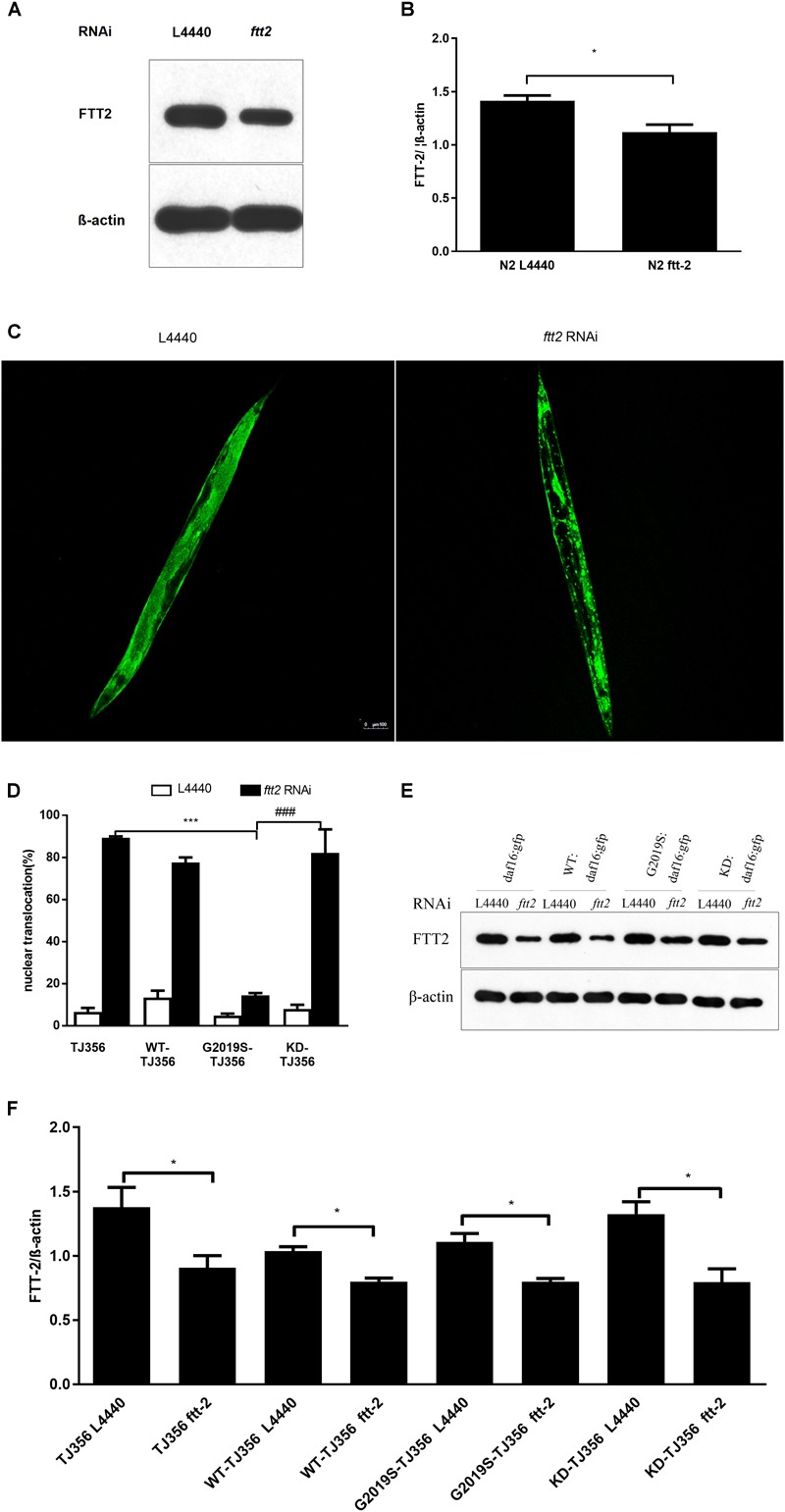
*C. elegansftt-2* is important in G2019S LRRK2-associated defect in DAF-16 nuclear translocation. Worms were synchronized and fed with control (L4440) RNAi or *ftt-2* RNAi. Worm lysate was followed by Western blotting with an antibody against pan 14-3-3 to detect FTT-2 protein (28 kDa). **(A)** Detection on Western blots of FTT-2 proteins (28 kDa) in N2 strain fed with control (L4440) RNAi or *ftt-2* RNAi. **(B)** Quantification to show the effect of *ftt2* RNAi. ^∗^*p* < 0.05. **(C)** Representative images show cytoplasmic localization (treated with control L4440 RNAi) and DAF-16 nuclear localization (treated with *ftt-2* RNAi). Scale bar = 100 μm. **(D)** Quantification of DAF-16 nuclear localization. Error bars indicate SEM. **(E)** Detection on Western blots of FTT-2 proteins (28 kDa) in *C. elegans* fed with control (L4440) RNAi or *ftt-2* RNAi. Worm lysate was followed by Western blotting with an antibody against pan-14-3-3. **(F)** Quantification to show the effect of *ftt2* RNAi. ^∗^*p* < 0.05, ^∗∗∗^*p* < 0.001 for TJ356 vs.G2019S TJ356; ###*p* < 0.001 for G2019S TJ356 vs. KD TJ356.

The *C. elegans* 14-3-3 protein ortholog FTT-2 acts as a key regulator of DAF-16 by binding to DAF-16 and regulating its sub-cellular localization via sequestration in the cytoplasm (Li et al., 2006). Given that 14-3-3 interacts with LRRK2, we investigated the role of 14-3-3 in G2019S LRRK2-associated inhibition of DAF-16 nuclear localization using RNA-mediated gene interference (RNAi) against *ftt-2*. Western blotting was performed to detect protein expression levels of strains N2 (Figures 3A,B) and TJ356, WT-TJ356, G2019S-TJ356, KD-TJ356 (Figures 3E,F) fed RNAi control OP50 or *ftt-2* OP50. DAF-16::GFP was diffusely and predominantly localized in the cytoplasm in the progeny of TJ356 receiving control RNAi (L4440), whereas DAF-16::GFP was predominantly localized in the nuclei of the TJ356 strain receiving *ftt-2* RNAi (Figure 3C). Furthermore, when worms were fed with control RNAi (L4440), all strains exhibited cytoplasmic localization of DAF-16. After being fed with *ftt-2* RNAi, worms from WT-TJ356 and KD-TJ356 strains exhibited DAF-16 nuclear localization. However, DAF-16 was still localized in the cytoplasm of the G2019S-TJ356 strain. The percentage of worms with nuclear translocation of DAF-16 was approximately the same among WT-TJ356, KD-TJ356 and TJ356 strains (L4440: TJ356, 6.4% ± 1.9%; G2019S-TJ356, 4.3% ± 1.2%; WT-TJ356, 13.4% ± 3.4%; KD-TJ356, 8.0% ± 2.0%; *ftt2* RNAi: TJ356, 88.0% ± 2.0%; G2019S-TJ356, 13.3% ± 1.9%; WT-TJ356, 77.5% ± 2.5%; KD-TJ356, 82.2% ± 11.2%). Compared with the TJ356 control strain, G2019S-TJ356 exhibited a significantly decreased number of nuclear DAF16::GFP (Figure 3D, *p* < 0.001); whereas, KD could rescue G2019S-mediated inhibition of DAF-16 nuclear translocation (Figure 3D, *p* < 0.001). Thus, our data suggest that G2019S inhibits *ftt-2* -induced DAF-16 nuclear localization.

### Human 14-3-3 β Protein Rescued G2019S LRRK2-Associated Toxicity in Response to Stress

**FIGURE 4:**
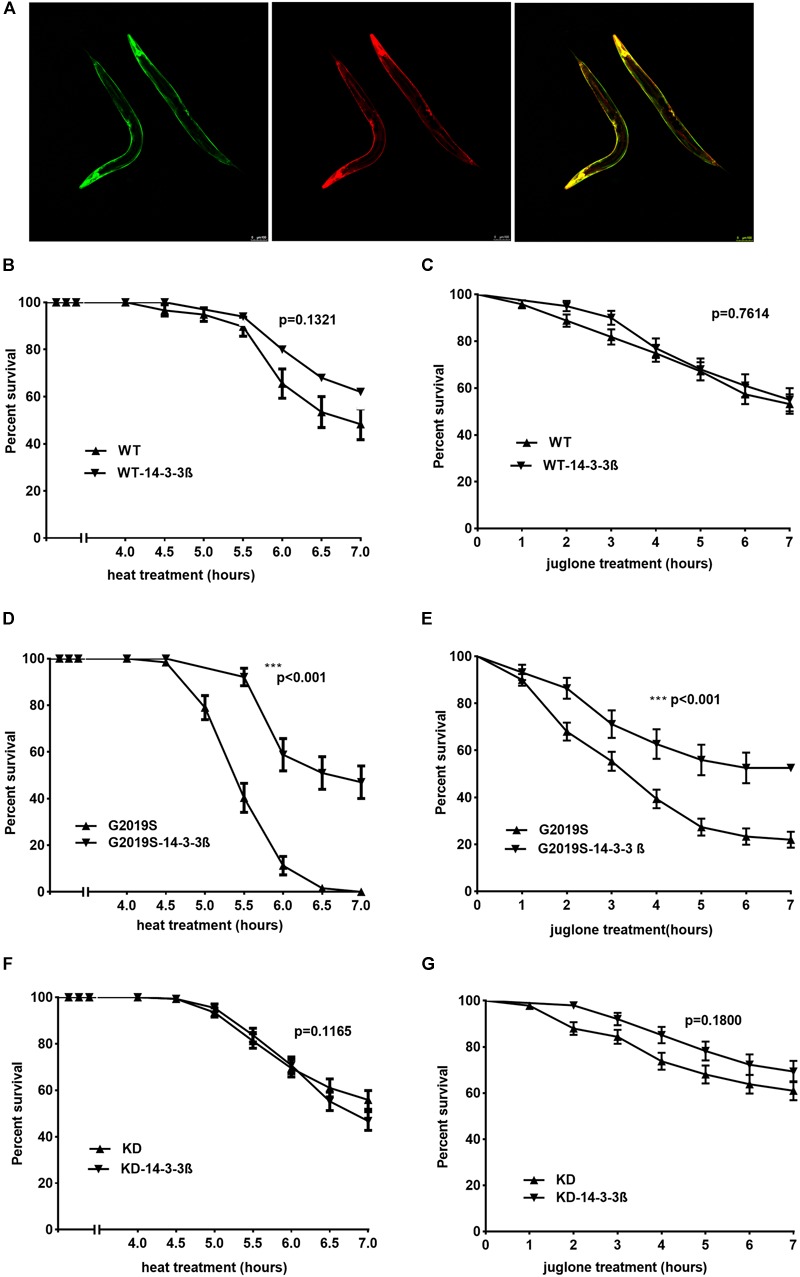
14-3-3 β protein can rescue G2019S LRRK2-associated toxicity in response to stress. **(A)** Representative images of adult worms pan-neuronal expressing human 14-3-3 β in LRRK2 transgenic worms visualized by epifluorescence microscopy (GFP fused with 14-3-3; RFP co-injection with LRRK2). Scale bar = 100 μm. **(B)** Human 14-3-3 β showed no effect on WT LRRK2 in response to heat stress. Error bars indicate SEM. *P* = 0.1321. **(C)** Human 14-3-3 β showed no effect on WT LRRK2 in response to juglone. Error bars indicate SEM. *P* = 0.7614. **(D)** Human 14-3-3 β can rescue G2019S LRRK2-associated toxicity in response to heat stress. Error bars indicate SEM. ^∗∗∗^*p* < 0.001. **(E)** Human 14-3-3 β can rescue G2019S LRRK2-associated toxicity in response to juglone. Error bars indicate SEM. ^∗∗∗^*p* < 0.001. **(F)** Human 14-3-3 β showed no effect on KD LRRK2 in response to heat stress. Error bars indicate SEM. *P* = 0.1165. **(G)** Human 14-3-3 β showed no effect on KD LRRK2 in response to juglone. Error bars indicate SEM. *P* = 0.1800.

To examine whether 14-3-3 can rescue G2019S LRRK2-associated toxicity, we generated *C. elegans* strains co-expressing human 14-3-3 β and LRRK2 by injecting a plasmid containing 14-3-3 β fused with a green fluorescent protein (GFP) marker into WT, G2019S and KD transgenic worms. Subsequently, WT-14-3-3 β, G2019S-14-3-3 β and KD-14-3-3 β were selected using the GFP marker (Figure 4A). After exposure to heat stress at 35°C for 7 h, all G2019S worms died, while survival rates were 48.3% ± 6.6% for WT, 62.0% ± 6.9% for WT-14-3-3 β, 47.1% ± 7.0% for G2019S-14-3-3 β, 55.8% ± 4.0% for KD, and 46.8% ± 4.0% for KD-14-3-3 β. The survival rate of G2019S was significantly different from that of G2019S-14-3-3 β (Figure 4D, *p* < 0.001), while the survival rate of WT (or KD) was similar to WT-14-3-3 β (or KD-14-3-3 β) (Figures 4B,F). In comparison, when worms were exposed to juglone for 7 h, survival rates were 53.1% ± 4.2% for WT, 55% ± 5.0% for WT-14-3-3 β, 22.0% ± 3.4% for G2019S, 52.5% ± 0.0% for G2019S-14-3-3 β, 61.0% ± 4.1% for KD, and 69.3% ± 4.6% for KD-14-3-3 β. The survival rate of G2019S was significantly different from that of G2019S-14-3-3 β (Figure 4E, *p* < 0.001), while the survival rate of WT (or KD) was similar to WT-14-3-3 β (or KD-14-3-3 β) (Figures 4C,G).

### Human 14-3-3 β Protein Rescued G2019S LRRK2-Mediated Inhibition of Stress-Resistance Gene mRNA Expression

**FIGURE 5:**
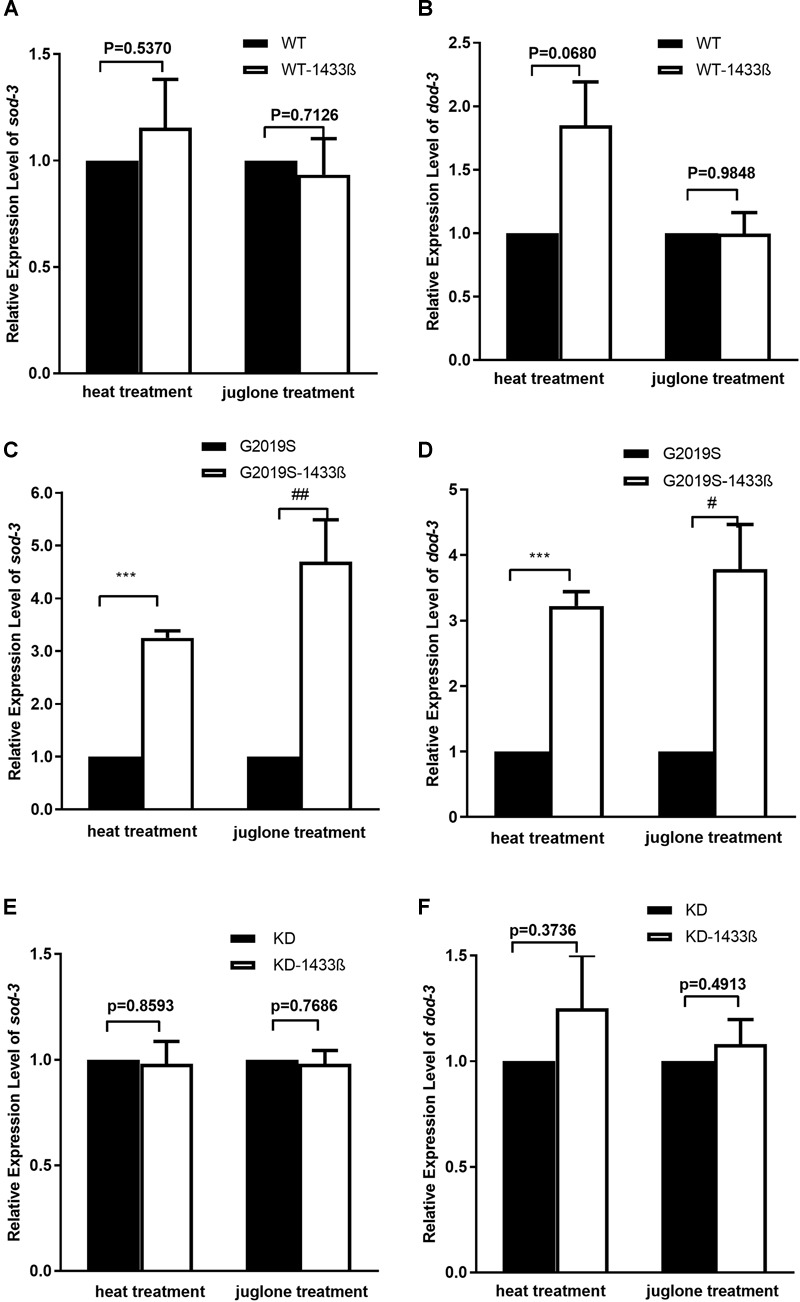
14-3-3 β protein can rescue G2019S LRRK2-associated defect in mRNA expression of stress-resistance genes. **(A)** Expression of *sod-3* in transgenic strains after heat treatment for 60 min. Human 14-3-3 β showed no effect on WT LRRK2 in mRNA expression of *sod-3* (*p* = 0.5370). Expression of *sod-3* in transgenic strains after juglone treatment for 60 min. Human 14-3-3 β showed no effect on WT LRRK2 in mRNA expression of *sod-3* (*p* = 0.7126). **(B)** Expression of *dod-3* in transgenic strains after heat treatment for 60 min. Human 14-3-3 β showed no effect on WT LRRK2 in mRNA expression of *dod-3* (*p* = 0.0680). Expression of *dod-3* in transgenic strains after juglone treatment for 60 min. Human 14-3-3 β showed no effect on WT LRRK2 in mRNA expression of *dod-3* (*p* = 0.9849). **(C)** Expression of *sod-3* in transgenic strains after heat treatment for 60 min. Human 14-3-3 β could rescue G2019S LRRK2-associated deficit in mRNA expression of *sod-3* (^∗∗∗^*p* < 0.001 versus G2019S strain). Expression of *sod-3* in transgenic strains after juglone treatment for 60 min. Nematodes expressing G2019S LRRK2 showed reduced expression of *sod-3* (^##^*p* < 0.01 versus RFP strain). **(D)** Expression of *dod-3* in transgenic strains after heat treatment for 60 min. Human 14-3-3 β could rescue G2019S LRRK2-associated deficit in mRNA expression of *dod-3* (^∗∗∗^*p* < 0.001 versus G2019S strain). Expression of *dod-3* in transgenic strains after juglone treatment for 60 min. Nematodes expressing G2019S LRRK2 showed reduced expression of *dod-3* (^#^*p* < 0.05 versus G2019S strain). **(E)** Expression of *sod-3* in transgenic strains after heat treatment for 60 min. Human 14-3-3 β showed no effect on KD LRRK2 in mRNA expression of *sod-3* (*p* = 0.8593). Expression of *sod-3* in transgenic strains after juglone treatment for 60 min. Human 14-3-3 β showed no effect on KD LRRK2 in mRNA expression of *sod-3* (*p* = 0.7686). **(F)** Expression of *dod-3* in transgenic strains after heat treatment for 60 min. Human 14-3-3 β showed no effect on KD LRRK2 in mRNA expression of *dod-3* (*p* = 0.3736). Expression of *dod-3* in transgenic strains after juglone treatment for 60 min. Human 14-3-3 β showed no effect on KD LRRK2 in mRNA expression of *dod-3* (*p* = 0.4913).

Our data show that co-expression of human *14-3-3 β* can rescue the expression of stress-resistant genes *sod-3* and *dod-3* in the G2019S transgenic strain in response to heat stress. When worms were exposed to heat stress at 35°C for 1 h, mRNA expression of *sod-3* and *dod-3* in the G2019S strain was significantly different from that of G2019S-14-3-3 β (Figures 5C,D, *p* < 0.001); whereas, the survival rate of WT (or KD) was similar to WT-14-3-3 β (or KD-14-3-3 β) (Figures 5A,B,E,F). Expression of *sod-3* in WT-14-3-3 β, G2019S-14-3-3 β, and KD-14-3-3 β was 0.88 ± 0.03, 0.92 ± 0.15, and 0.88 ± 0.12, respectively; whereas, *dod-3* expression was 1.41 ± 0.13, 1.16 ± 0.08, and 1.25 ± 0.13, respectively.

As shown in Figure 5, human 14-3-3β can rescue expression of stress-resistant genes *sod-3* and *dod-3* in the G2019S transgenic strain in response to juglone. When worms were exposed to juglone for 1 h, mRNA expression of *sod-3* and *dod-3* in the G2019S strain was significantly different from that of G2019S-14-3-3 β (Figures 5C,D, *p* < 0.01 for *sod-3* and *p* < 0.05 for *dod-3*); whereas, the survival rate of WT (or KD) was similar to WT-14-3-3 β (or KD-14-3-3 β) (Figures 5A,B,E,F). Expression of *sod-3* in WT-1433 β, G2019S-1433 β, and KD-1433 β was 1.02 ± 0.04, 1.07 ± 0.01, and 1.05 ± 0.02, respectively; whereas, *dod-3* expression was 1.05 ± 0.16, 1.21 ± 0.09and 1.07 ± 0.11, respectively.

## Discussion

In the present study, we examined the role of 14-3-3 and DAF-16 proteins in LRRK2 pathophysiology in *C. elegans*. Our data is consistent with our hypothesis that G2019S LRRK2 increased sensitivity to stress and impaired stress-induced DAF-16 nuclear translocation. Furthermore, G2019S LRRK2 inhibited 14-3-3 protein-associated DAF-16 nuclear translocation. In contrast, overexpression of human 14-3-3 β could rescue G2019S LRRK2-associated toxicity in response to stress and ameliorate G2019S LRRK2-associated down-regulation of *sod-3* and *dod-3* expression (two DAF-16 pathway genes). Collectively, the present data suggest that increased stress susceptibility of G2019S LRRK2 is associated with inhibition of DAF-16 nuclear translocation in a 14-3-3-associated manner. Given the critical role of DAF-16 in stress resistance, our findings imply that DAF-16 may play an important role in 14-3-3-mediated attenuation of G2019S LRRK2 toxicity.

### G2019S Increases Sensitivity to Stress by Impairing Nuclear Localization of DAF-16

We generated transgenic *C. elegans* expressing human WT LRRK2 and PD-linked mutant G2019S LRRK2 or G2019S kinase dead LRRK2 in all neurons. Consistent with previous studies, sensitivity to stress was significantly increased in G2019S worms.

DAF-16, the only FoxO transcription factor in *C. elegans*, plays an important role in stress resistance. DAF-16 is activated by nuclear translocation in response to environmental stress. Once DAF-16 is activated, it activates expression of downstream genes such as *sod-3* and *dod-3* to increase stress resistance. Consistently, under heat and juglone stress, nuclear translocation of DAF-16 was evident in most *C. elegans* of both control and WT strains; whereas, DAF-16 was only detected in a relatively small population of G2019S *C. elegans*. Furthermore, G2019S-induced impairment of DAF-16 nuclear translocation resulted in loss of stress resistance, suggesting that blockage of DAF-16 nuclear translocation may be one mechanism by which G2019S elicits toxicity.

### G2019S Impairs 14-3-3 Knockdown-Induced DAF-16 Nuclear Translocation

Previously, LRRK2 has been shown to directly phosphorylate FoxO, an analog of DAF-16, initiating an apoptotic pathway (Kanao et al., 2010). Phosphorylation of DAF-16 is a key upstream event of DAF-16 signaling. Following phosphorylation, DAF-16 can translocate to the nucleus to activate downstream gene expression. The 14-3-3 family of proteins is a major player in the regulation of DAF-16 translocation between the nucleus and cytoplasm. For example, the 14-3-3 protein FTT-2 has been reported to bind to Akt-phosphorylated DAF-16 and consequently confine it to the cytoplasm. However, inhibition of 14-3-3 protein by either genetics or stress (oxidative or heat stress) (Oh et al., 2005; Sunayama et al., 2005; Landis and Murphy, 2010) promotes nuclear translocation of DAF-16. Consistently, in the present study, 14-3-3 knockdown by siRNA induced DAF-16 nuclear translocation. Furthermore, G2019S blocked, whereas G2019S kinase dead completely restored, 14-3-3 knockdown-induced nuclear translocation of DAF-16. Collectively, our data suggest that G2019S blocked 14-3-3 knockdown-induced nuclear translocation of DAF-16 in a kinase-dependent manner.

### 14-3-3 β Ameliorates G2019S LRRK2-Induced Toxicity by Restoring *sod-3* and *dod-3*

14-3-3 proteins are a key hub for dysregulated proteins in transcriptional analysis of PD patients (Ulitsky et al., 2010). All 14-3-3 isoforms except 14-3-3 σ can interact with the PD-related protein LRRK2 (Nichols et al., 2010). Our results showed that overexpression of human 14-3-3 β could rescue G2019S LRRK2-associated deficiency in response to stress. Additionally, this rescue effect was associated with restored expression of *sod-3* and *dod-3* in G2019S worms. Given that *sod-3* and *dod-3* are direct downstream genes of DAF-16, our results suggest that 14-3-3 may rescue G2019S-mediated loss of stress resistance via modulation of DAF-16 activity.

### Potential Interplay Among LRRK2, 14-3-3, and DAF-16

Phosphorylation of DAF-16 is a key event in DAF-16 signaling pathways. Phosphorylation can promote or inhibit DAF-16 nuclear translocation depending on the function of kinases. For example, phosphorylation by Akt restricts DAF-16 to the cytoplasm, while JNK promotes nuclear translocation (Oh et al., 2005).

We found that G2019S LRRK2 could block stress/14-3-3 knockdown-associated nuclear translocation. This event is kinase-dependent, as the kinase dead strain of G2019S completely rescued this event. Given that LRRK2 can directly interact with 14-3-3 and DAF-16, both interactions may have a role in nuclear translocation of DAF-16. Interestingly, pathogenic mutations such as R1441C, R1441G, R1441H, Y1699C, and I2020T, but not G2019S, disrupt the interaction with 14-3-3 (Nichols et al., 2010). Furthermore, oxidative stress reportedly reduces the binding of 14-3-3 to WT, kinase-dead LRRK2 and G2019S to the same degree (Mamais et al., 2014). Thus, the interaction of G2019S with 14-3-3 is less likely to play a critical role in DAF-16 nuclear translocation.

**FIGURE 6:**
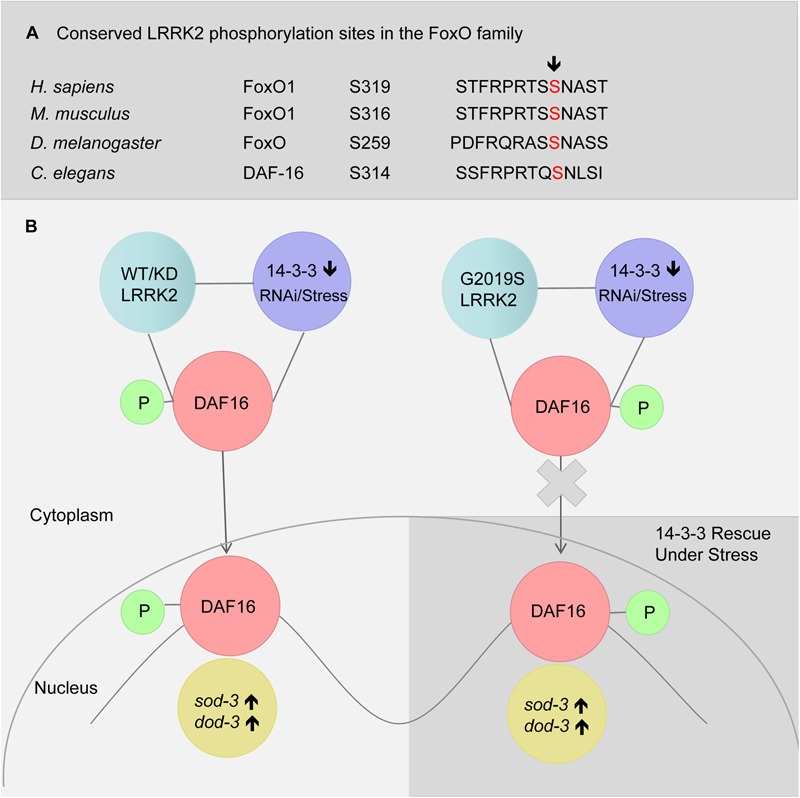
A model of LRRK2 and 14-3-3 associated DAF-16 nuclear translocation in response to stress. **(A)** Alignment of putative LRRK2 phosphorylation sites in FoxO family. **(B)** RNAi knockdown of 14-3-3 or stress (heat or juglone treatment) induced DAF-16 nuclear translocation in *C. elegans* expressing WT-LRRK2 or G2019S-KD LRRK2. Meanwhile, G2019S LRRK2 inhibited DAF-16 nuclear translocation, resulting in stress response deficiency which was rescued by 14-3-3.

LRRK2 has been reported to phosphorylate FoxO1 at S319 (Kanao et al., 2010). We also identified S314 to be a conserved phosphorylated site in DAF-16 (Figure 6A). In the present study, G2019S did not affect sub-cellular localization of DAF-16 under normal conditions (data not shown). We propose that G2019S LRRK2 phosphorylation of DAF-16 inhibits nuclear translocation under normal conditions. There may be two possible explanations for this observation. First, phosphorylation of DAF-16 by G2019S alone may not be sufficient to induce the translocation of DAF-16. Alternatively, G2019S-mediated phosphorylation of DAF-16 may restrict DAF-16 to the cytoplasm. In the current study, we found that G2019S impaired 14-3-3 knockdown-induced DAF-16 translocation, suggesting that the second possibility is the most likely scenario. Normally in WT and G2019S-KD LRRK2, LRRK2 binds with DAF-16 following its phosphorylation to restrict DAF-16 to the cytoplasm. Under stress and 14-3-3 knockdown, 14-3-3 may interact with additional kinases such as JNK to further promote DAF-16 nuclear translocation (Figure 6B). In the case of G2019S LRRK2, G2019S possesses increased kinase activity in relation to LRRK2; thus, under stress or 14-3-3 knockdown, G2019S negatively regulates DAF-16 to prevent nuclear translocation. Further study is needed to examine whether G2019S can indeed phosphorylate specific sites of DAF-16 to directly restrict DAF-16 nuclear translocation or indirectly via reduce the interaction between DAF-16 and 14-3-3. Finally, given the complex regulatory mechanisms of DAF-16 translocation, other possibilities such as varying phosphorylation levels, roles for additional kinases including Akt and JNK, potential involvement of Sir2, and post-translational modifications also need further investigation.

## Materials and Methods

### Strains

Several *C. elegans* strains were obtained from the Caenorhabditis Genetics Center or elsewhere, as indicated, including Bristol N2, TJ356, and BZ555 strains. All strains used were maintained and handled as previously described (Brenner, 1974). Strains were maintained at 20°C on NGM plates with OP50 as food, unless otherwise noted. Bristol N2 was used as a reference strain. NGM was made with Agar (MBCHEN) and Tryptone (OXOID, Ltd.) and other chemicals from Guangzhou Chemical Reagent Factory. Mixed-stage animals were maintained as bulk culture on NGM agar at room temperature (20°C). For most experiments, *C. elegans* strains were age synchronized using a bleaching method, and underwent development from larval stage L1 to L4, which was considered as the beginning of adulthood and counted as adult day 0. L4-stage worms were then picked and transferred to new NGM plates for growth into adults.

### Plasmids

Human cDNA encoding full-length FLAG-tagged human WT LRRK2 and mutants (LRRK2 G2019S and G2019S KD) generated by site-directed mutagenesis were previously constructed in pcDNA3.1 vectors, as described (Smith et al., 2005). The *C. elegans* promoter for the pan-neuronal transporter UNC51 was used to drive transgene expression in all neurons. The pu51p vector including unc51 promoter was obtained from Kuwahara et al. (2008). The pu51p vector was used as a *C. elegans* expression vector. We constructed transgenic plasmids expressing human WT LRRK2 and mutants (G2019S and G2019S KD) with the pu51p vector. WT LRRK2 and mutant (G2019S and G2019S KD) LRRK2 cDNA fragments were obtained by cutting between *NheI* and *BamHI* sites of modified pcDNA3.1 and inserting fragments into pu51p between *NheI* and *KpnI* sites of pu51p. Additionally, we constructed transgenic a pu51p-14-3-3 β-GFP plasmid expressing 14-3-3 β protein fused with eGFP. First, the enhanced GFP sequence was inserted into *XhoI* and *SacI* sites of pu51p. Second, the PCR product of 14-3-3 β was inserted into *NheI* and *XhoI* sites immediately upstream of GFP in the modified pu51p to create pu51p-14-3-3-GFP. All RNAi vectors were purchased from Open Biosystems (Boston, MA, United States).

### Generating Transgenic *C. elegans* Strains

LRRK2 transgenic nematodes were created by co-injecting a cocktail of DNA containing 50 ng/μL of pu51p-WT LRRK2, pu51p-G2019S LRRK2, or pu51p-G2019S KD LRRK2 plasmid along with the co-marker pu51p-RFP (100 ng/μL). Human *14-3-3 β* transgenic nematodes were created by injecting 100 ng/μL of pu51p-14-3-3β-GFP plasmid. This cocktail of DNA was injected into the gonad of young adult hermaphrodites of the N2 strain using a previously described method (Mello et al., 1991). Injected nematodes were grown at 20°C. Nematodes with stable arrays (over 50% transmittance) were analyzed by PCR for the presence LRRK2 cDNA and chosen for chromosomal integrations. L4 nematodes carrying arrays were subjected to UV-irradiation (30,000 μJ/cm^2^) and grown further to generate F1 progenies. Two hundred F1 nematodes were individually grown in a single plate and selected for 75% transmittance. Four nematodes were picked from every F2 population with 75% transmittance, and then individually grown in single plate and selected for 100% transmittance. With a yield of 1–2% stable integrants, transgenic nematodes were out-crossed with Bristol N2 nematodes at least five times and PCR analyzed for the presence of transgenic cDNA. LRRK2/DAF-16::GFP strains (carrying GFP) were created by conventional crossing of WT LRRK2 or mutant LRRK2 strains with TJ356 (DAF-16::GFP), and verified by checking both RFP and GFP markers. Representative integrated strains from each strain were used in repeated experiments throughout this study, and their detailed genotypes and strain designations are listed in Table 1.

**Table 1:** Transgenic *C. elegans* strains used in this study.

Designation	Genotype
1. RFP	*Punc51p::RFP*
2. WT	*Punc51p::LRRK2(WT)*
3. G2019S	*Punc51p::LRRK2(G2019S)*
4. KD	*Punc51p::LRRK2(G2019S D1994A)*
5. TJ356	*zIs356 IV [DAF-16p::DAF-16a/b::GFP + rol-6]*
6. WT-TJ356	*Punc51p::LRRK2(WT);zIs356 IV*
7. G2019S-TJ356	*Punc51p::LRRK2(G2019S);zIs356 IV*
8. KD-TJ356	*Punc51p::LRRK2(G2019S D1994A);zIs356 IV*
9. WT-14-3-3β	*Punc51p::LRRK2(WT);Punc51p::14-3-3β::GFP*
10. G2019S-14-3-3β	*Punc51p::LRRK2(G2019S);Punc51p::14-3-3β::GFP*
11. KD-14-3-3β	*Punc51p::LRRK2(G2019S D1994A);Punc51p::14-3-3β::GFP*

Nematodes were synchronized either by a bleaching method or letting nematodes lay eggs for 2–3 h.

### Stress Assay

Assays were performed using two dishes of 25 adult nematodes for each condition and strain. Nematodes were exposed to NGM agar containing 400 μM juglone or heated to 35°C. Treatment with juglone was begun on adult day 3, while treatment at 35°C was begun on adult day 2. Nematode viability was checked every hour with a soft touch at the tip of pharynx of the nematode. Nematodes that crawled up the side of the plate were excluded from all analyses.

### RNAi Knockdown

RNAi knockdown assay was performed according to Kamath et al. (2001). HT115 bacteria was grown overnight in LB media containing 50 μg/mL ampicillin. Bacteria was then plated on NGM plates containing 1 mM IPTG and allowed to grow overnight at room temperature. Nematodes were synchronized by a bleaching method and exposed at L4 stage to HT115 bacteria containing either the pL4440 expression vector with the *ftt2* open reading frame (ORF) (Open Biosystems) or empty vector. On adult day 2 of the next generation, nematodes were used in several experiments.

### DAF-16 Nuclear Localization Assay

For quantification of DAF-16::GFP localization, synchronized eggs from TJ356 animals (i.e., transgenic animals expressing DAF-16::GFP) or other strains (as indicated) were seeded onto either control or appropriate RNAi plates. For stress response experiments, adult day 1 worms were transferred to new plates and subjected to heat shock (35°C) or plates containing 400 μM juglone. GFP localization was then analyzed using a Nikon AZ100 fluorescent microscope at 5× magnification. An animal was scored as having nuclear GFP if more than one head hypodermic nuclei contained DAF-16::GFP. For single time point experiments, worms were blindly scored for the presence or absence of GFP accumulation within the nuclei of indicated cells (*n* ≥ 120 or greater).

### Western Blotting

Protein extracts were prepared from mixed-stage worms grown to near confluence on five 60-mm NGM plates. Worms were washed with M9 buffer and collected by brief centrifugation at 3,000 × *g* for 1 min. The worm pellet was homogenized in ice-cold lysed buffer (1% Triton X-100 in PBS) supplemented with a cocktail of protease inhibitors (Roche), and then sonicated five times for 5 s each. The mixture was then incubated on ice for 30 min. Homogenates were clarified after centrifugation at 14,000 × *g* for 20 min. The supernatant was collected for sodium dodecyl sulfate polyacrylamide gel electrophoresis (SDS-PAGE) and Western blotting using anti-14-3-3 (1:8000, Abcam) for FTT-2 detection. An anti-actin antibody (1:4000, PTG) was used for protein control.

### Quantitative RT Real-Time PCR Assay

Total RNA from *C. elegans* was extracted using an E.Z.N.A. total RNA extraction kit (OMEGA Biotek). Intact RNA was checked by running a 1.0% agarose/formaldehyde gel and quantified spectrometrically (Beckman Coulter DU 800) before proceeding to subsequent steps. Five-hundred nanograms of total RNA were reverse-transcribed using PrimeScript^TM^ RT Master Mix (Perfect Real Time) Kit (Takara) according to the manufacturer’s instructions. Real-Time PCR was performed on an Opticon MONITORTM Software (MJ Research) using SYBR^®^ Premix Ex Taq^TM^ II (TliRNaseH Plus) (Takara). Expression levels for each target gene were calculated by the 2^-ΔΔCT^ method (Livak and Schmittgen, 2001). All analyses were performed in triplicate. Primers used for RT real-time PCR are listed in Table 2.

**Table 2:** Amplimer sets used for PCR.

Name	Forward sequence	Reverse sequence
*act-1*	5′-CACGGTATCGTCACCAACTG-3′	5′-GCTTCAGTGAGGAGGACTGG-3′
*sod-3*	5′-TCGGTTCCCTGGATAACTTG-3′	5′-TTCCAAAGGATCCTGGTTTG-3′
*dod-3*	5′-GCCATGTGCATATTGTGGAG-3′	5′-AGGAGGACGTATCCGATGAA-3′

### Statistical Analysis

Survival analysis was done using logrank test. According to reference and previous study, we need 50 animals per group. Statistical analysis was done using SPSS Statistics software. Survival curves were analyzed by the Kaplan–Meier method. Chi-square was conducted in the daf16 translocation assay. We take Convenience Sampling. At least 50 animals were randomly selected in stress assay and 30 animals were randomly selected in daf16 translocation assay. Descriptive statistics of the results are using mean and SD. Results of Western and qRT-PCR were evaluated by using a *t*-test. All the curves and column diagrams were performed using Graphpad Prism 7 software.

## Author Contributions

SL and ZP conceived and coordinated the study and wrote the paper. SL, FS, and WG designed, performed, and analyzed the experiments shown in Figures 1, 2, 4, 5. SL, YZ, and SH designed, performed, and analyzed the experiments shown in Figures 3, 6. JZ, CR, and E-KT provided technical assistance and contributed to the preparation of the figures. All authors reviewed the results and approved the final version of the manuscript.

## Conflict of Interest Statement

The authors declare that the research was conducted in the absence of any commercial or financial relationships that could be construed as a potential conflict of interest.

## Supplementary Material

FIGURE S1 Generation of *C. elegans* lines over expressing WT or G2019S or G2019S kinase dead (KD) LRRK2. **(A)** Representative images of adult worms from LRRK2 transgenic lines visualized by epifluorescence microscopy (RFP) of the whole body. Scale bar = 50 μm. **(B)** Detection of transgene LRRK2. Single worm PCR is detected for a ∼617 bp fragment of LRRK2 cDNA. Plasmid WT LRRK2 used for transformation served as a positive control. M: DNA size marker in kb. **(C)** Detection on Western blots of LRRK2 proteins (280 kDa) in *C. elegans* over expressing WT, G2019S, and KD LRRK2, but not in the RFP strain. Worm lysate was followed by Western blotting with an antibody against FLAG.

FIGURE S2 DA neurodegeneration in *C. elegans* lines over expressing WT or G2019S or G2019S kinase dead (KD) LRRK2. **(A)** G2019S LRRK2 expression leads to DA neurodegeneration. Control and *C. elegans* expressing WT or KD LRRK2 contained intact DA neurons (CEP neurons) and smooth neuritis, whereas the G2019S LRRK2 over expression line displayed prominent loss of DA neurons (white arrows) during adult day 2. Scale bar = 75 μm. **(B)** The DA neuron degeneration was quantified by the loss of CEP neurons in transgenic animals during adult day 2. BZ555 (Pdat-1::GFP) worm was a control. Error bars indicate SEM. G2019S LRRK2 strain and control were significantly different (*p* < 0.01) whereas KD could rescue G2019S-mediated loss of DA neurons. **(C)** Numbers of bends every 30 s for control and transgenic lines in adult day 2. G2019S LRRK2 strain and control were significantly different (*p* < 0.001) whereas KD could rescue G2019S-mediated loss of motor ability. WT LRRK2 strain was similar with control strain.

TABLE S1 Transgenic *C. elegans* strains used in DA neuron degeneration assay.
