# Supplementary figures and images for: G2019S LRRK2 Increases Stress Susceptibility Through Inhibition of DAF-16 Nuclear Translocation in a 14-3-3 Associated-Manner in Caenorhabditis elegans
Source: Front Neurosci. 2018 Nov 7;12:782. doi: 10.3389/fnins.2018.00782 (PMC6234837; doi:10.3389/fnins.2018.00782)

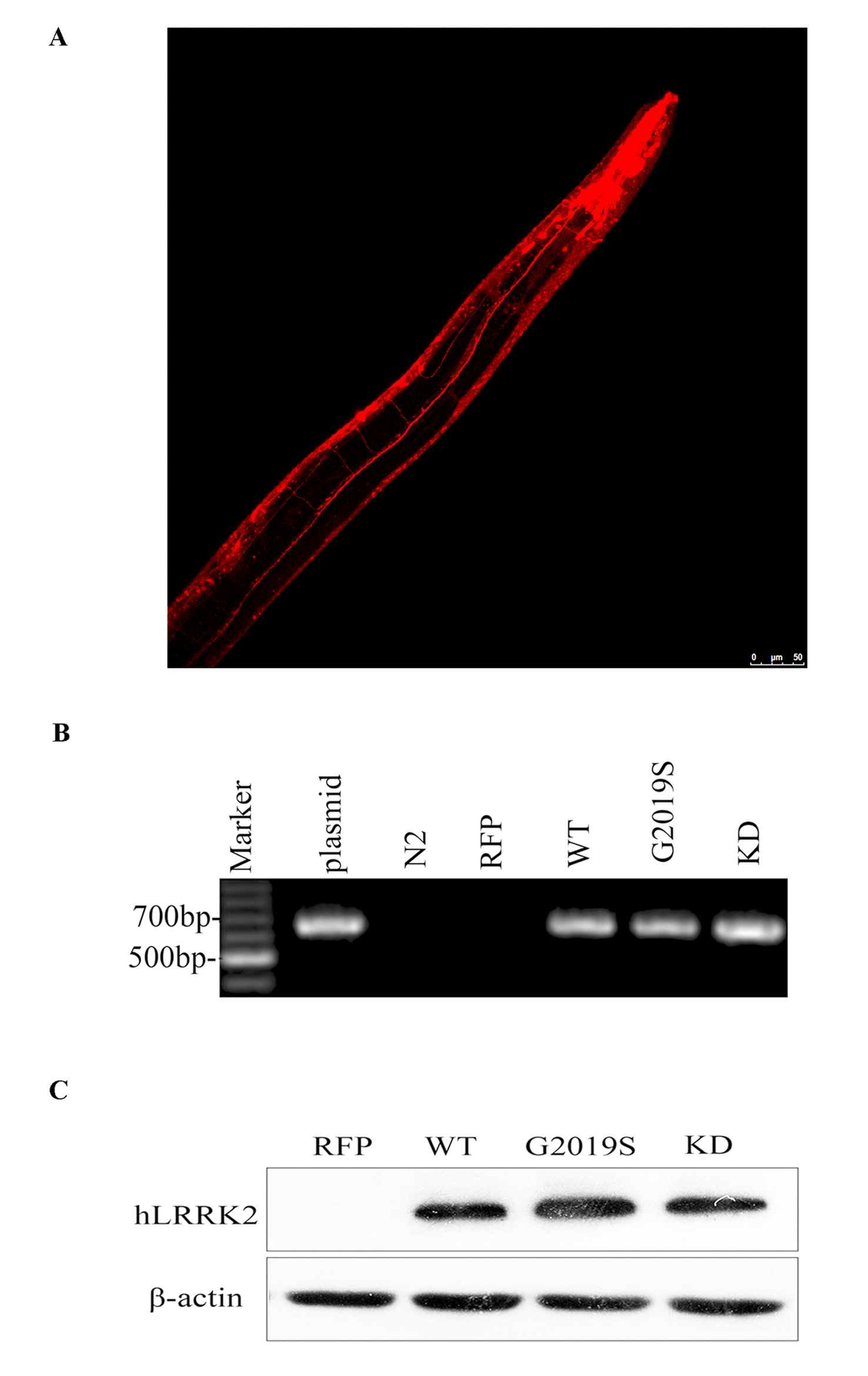

Supplement: FIGURE S1 — Generation of C. elegans lines over expressing WT or G2019S or G2019S kinase dead (KD) LRRK2. (A) Representative images of adult worms from LRRK2 transgenic lines visualized by epifluorescence microscopy (RFP) of the whole body. Scale bar = 50 μm. (B) Detection of transgene LRRK2. Single worm PCR is detected for a ∼617 bp fragment of LRRK2 cDNA. Plasmid WT LRRK2 used for transformation served as a positive control. M: DNA size marker in kb. (C) Detection on Western blots of LRRK2 proteins (280 kDa) in C. elegans over expressing WT, G2019S, and KD LRRK2, but not in the RFP strain. Worm lysate was followed by Western blotting with an antibody against FLAG. [file Image_1.TIF]

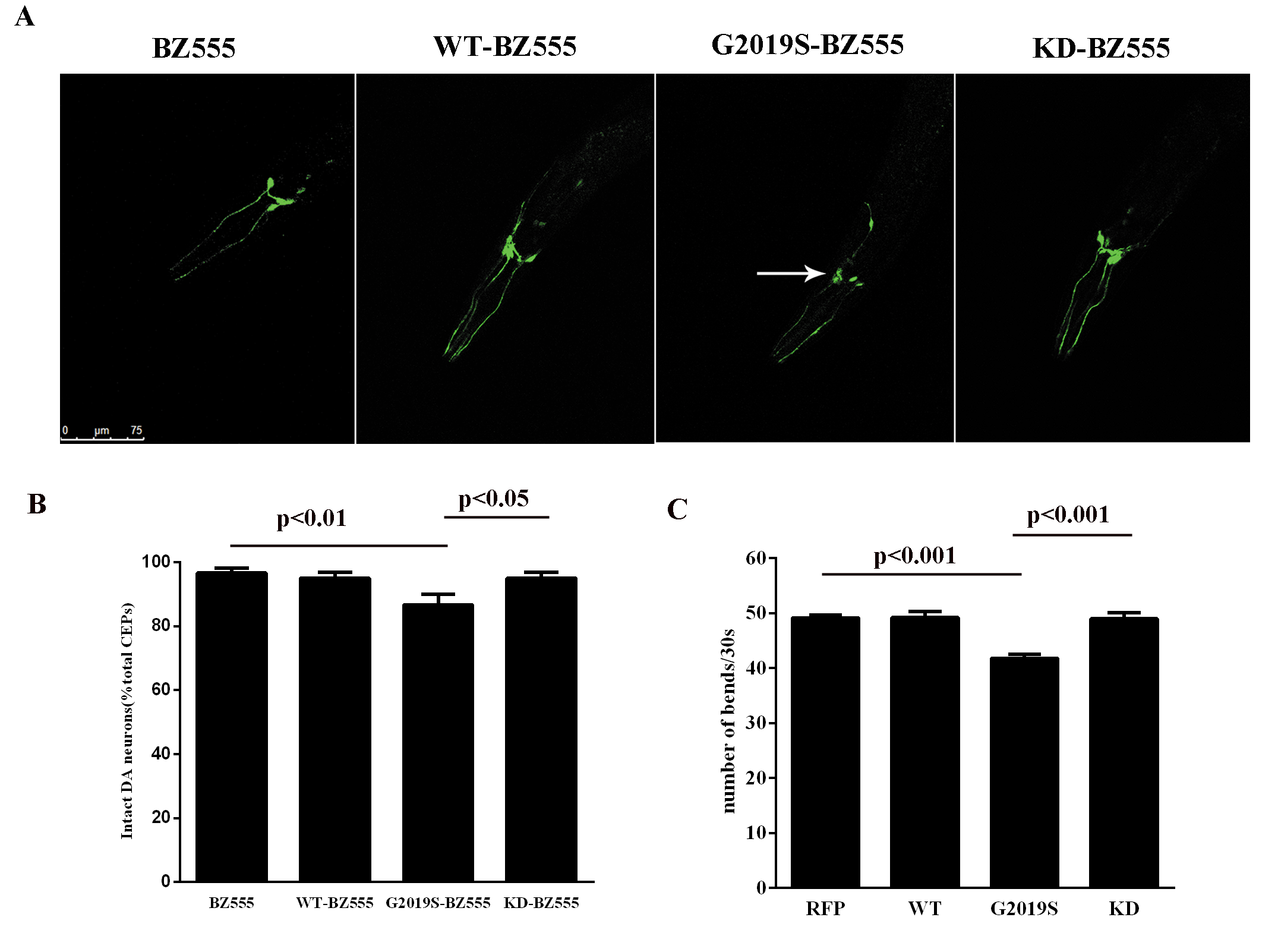

Supplement: FIGURE S2 — DA neurodegeneration in C. elegans lines over expressing WT or G2019S or G2019S kinase dead (KD) LRRK2. (A) G2019S LRRK2 expression leads to DA neurodegeneration. Control and C. elegans expressing WT or KD LRRK2 contained intact DA neurons (CEP neurons) and smooth neuritis, whereas the G2019S LRRK2 over expression line displayed prominent loss of DA neurons (white arrows) during adult day 2. Scale bar = 75 μm. (B) The DA neuron degeneration was quantified by the loss of CEP neurons in transgenic animals during adult day 2. BZ555 (Pdat-1::GFP) worm was a control. Error bars indicate SEM. G2019S LRRK2 strain and control were significantly different (p < 0.01) whereas KD could rescue G2019S-mediated loss of DA neurons. (C) Numbers of bends every 30 s for control and transgenic lines in adult day 2. G2019S LRRK2 strain and control were significantly different (p < 0.001) whereas KD could rescue G2019S-mediated loss of motor ability. WT LRRK2 strain was similar with control strain. [file Image_2.TIF]
