# Supplementary material for: G2019S LRRK2 Increases Stress Susceptibility Through Inhibition of DAF-16 Nuclear Translocation in a 14-3-3 Associated-Manner in Caenorhabditis elegans
Source: Front Neurosci. 2018 Nov 7;12:782. doi: 10.3389/fnins.2018.00782 (PMC6234837; doi:10.3389/fnins.2018.00782)
Supplement: TABLE S1 — Transgenic C. elegans strains used in DA neuron degeneration assay. [file Table_1.DOC]

S1 Table Transgenic *C.elegans* strains used in this study

| Designation | Genotype |
| --- | --- |
| 1.BZ555 | *egIs1 [dat-1p::GFP]* |
| 2. WT-BZ555 | *Punc51p::LRRK2(WT);egIs1* |
| 3.G2019S-BZ555 | *Punc51p::LRRK2(G2019S);egIs1* |
| 4.KD-BZ555 | *Punc51p::LRRK2(G2019S D1994A);egIs1* |
